# A 2-Year Longitudinal Randomized Control Trial of Speed of Processing Cognitive Training in Aging Adults with HIV-Associated Neurocognitive Disorder: Results of the Think Fast Study

**DOI:** 10.1007/s10461-024-04409-9

**Published:** 2024-07-01

**Authors:** David E. Vance, Pariya L. Fazeli, Andres Azuero, Jennifer S. Frank, Virginia G. Wadley, James L. Raper, Caitlin N. Pope, Karlene K. Ball

**Affiliations:** 1 School of Nursing, University of Alabama at Birmingham, Birmingham, AL 35294-1210 USA; 2 School of Medicine, University of Alabama at Birmingham, Birmingham, AL USA; 3 The 1917 (HIV/AIDS) Clinic, School of Medicine, University of Alabama at Birmingham, Birmingham, AL USA; 4 Department of Health, Behavior & Society, University of Kentucky, Lexington, KY USA; 5 Department of Psychology, UAB, University of Alabama at Birmingham, Birmingham, AL USA

**Keywords:** AIDS, Cognitive remediation therapy, Cognitive impairment, Brain fitness, Cognitive reserve, Cognitive training, Neuroplasticity, SIDA, Terapia de remediación cognitive, Deterioro cognitive, Aptitud cerebral, Reserva cognitive, Entrenamiento cognitive, Neuroplasticidad

## Abstract

Nearly 40% of people with HIV (PWH) experience HIV-associated Neurocognitive Disorder (HAND). In this 3-group efficacy study, 216 PWH 40 + years with HAND or borderline HAND were randomized to either: (1) 10 h of SOP training (*n* = 70); (2) 20 h of SOP training (*n* = 73), or (3) 10 h of Internet navigation training (*n* = 73; contact control group). Participants were administered a measure of SOP [i.e., the Useful Field of View Test (UFOV®)] at baseline, at posttest immediately after training, and at year 1 and year 2 follow up. Intent-to-treat linear mixed-effect models with subject-specific intercept and slope were fitted to estimate between-group mean differences at the follow-up time-points. At the post-intervention time-point, small beneficial SOP training effects were observed for the 10-h group in UFOV® total (*d* = 0.28, *p* = 0.002). Effects were of larger magnitude for the 20-h group in these same outcomes [UFOV® total (*d* = 0.43, *p* < 0.001)]. These results indicated better benefit with more training. No intervention effect was observed at year 1. At year 2, beneficial effects of small magnitude were observed again in the 10-h group [UFOV® total (*d* = 0.22, *p* = 0.253)] with larger small-to-moderate magnitude in the 20-h group [UFOV® total (*d* = 0.32, *p* = 0.104)]. This study suggests that SOP training can improve a key indicator of this cognitive performance and that treatment gains are small-to-moderate over a two-year period. Prior literature suggests slower SOP is predictive of impairment in everyday functioning in older PWH; such an approach could potentially improve everyday functioning in PWH.

**Supplementary Information:**

The online version contains supplementary material available at 10.1007/s10461-024-04409-9.

## Abstracto

Cerca del 40% de las personas viviendo con VIH (PVV) experimentan Trastorno Neurocognitivo Asociado al VIH (HAND, por sus siglas en inglés). En este estudio de eficacia de 3 grupos, se aleatorizó a 216 PVV mayores de 40 años de edad con HAND o HAND límite a: (1) 10 horas de entrenamiento en velocidad de procesamiento (SOP, por sus siglas en inglés) (*n* = 70); (2) 20 horas de entrenamiento SOP (*n* = 73), o (3) 10 horas de entrenamiento en navegación por Internet (*n* = 73; grupo control de contacto). Se administró una medida de SOP a los participantes [la Prueba de Campo de Visión Útil (UFOV®)] al inicio, inmediatamente después del entrenamiento, y en el seguimiento de año 1 y año 2. Los datos se analizaron bajo el principio de intención de tratar, utilizando modelos lineales de efectos mixtos para estimar las diferencias promedio entre grupos en los puntos de seguimiento. En el punto de tiempo de post- entrenamiento, se observaron pequeños efectos beneficiosos del entrenamiento SOP para el grupo de 10 horas en el puntaje total de UFOV® (*d* = 0.28, *p* = 0.002). Para esta misma medida, los efectos fueron de mayor magnitud en el grupo de 20 horas [UFOV® total (*d* = 0.43, *p* < 0.001)]. Estos resultados indicaron un mayor beneficio con más entrenamiento. No se observó ningún efecto de intervención en el año 1. En el año 2, se observaron efectos beneficiosos de pequeña magnitud nuevamente en el grupo de 10 horas [UFOV® total (*d* = 0.22, *p* = 0.253)] y en el grupo de 20 horas [UFOV® total (*d* = 0.32, *p* = 0.104)] con una magnitud pequeña a moderada). Este estudio confirma que el entrenamiento SOP puede mejorar un indicador clave de este rendimiento cognitivo y que las ganancias del tratamiento son pequeñas a moderadas durante un período de dos años. La literatura previa sugiere que una SOP más lenta es predictiva de deterioro en el funcionamiento diario en PVV mayores; tal enfoque podría mejorar potencialmente el funcionamiento diario en PVV.

Approximately 40% of people with HIV (PWH) develop cognitive impairments along a spectrum of HIV-associated neurocognitive disorder (HAND) ranging from mild to severe [1]. Such cognitive impairments can interfere with everyday functioning and reduce quality of life [1–4] Currently, nearly 51% of PWH in the U.S. are aged 50 years and older [5], and by 2030 this demographic is projected to swell to 70% [6]. To prevent and remediate such cognitive impairments in PWH as they age, interventions are needed [7].

Computerized cognitive training programs have been applied across non-pathological [8] as well as in many cognitively-vulnerable populations such as HIV [9]. Studies on computerized cognitive training provide several insights that inform future directions in aging PWH [10]. First, these studies demonstrate, including a study in PWH [11], that it may be more feasible and effective to improve performance by targeting a specific cognitive domain rather than trying to change overall cognitive performance [11]. This domain-specificity was confirmed in a systematic review of 13 computerized cognitive training studies in PWH, in which it was found that most interventions produced a positive improvement in the cognitive domain that was targeted for training (e.g., memory training improved memory functioning) [9]. Second, prior studies on cognitive training demonstrate that speed of processing (SOP) may be a cognitive domain that is very amenable by cognitive training [8]. A meta-analysis of 52 cognitive training studies in seronegative community-dwelling older adults 60–82 years old found that significant cognitive improvement was highest in SOP (*g* = 0.31) [8]. Similarly, in one of the largest randomized controlled trials of cognitive training to date (*N* > 2000), the ACTIVE (Advanced Cognitive Training for Independent and Vital Elderly) study found that older adults completing 10 h of SOP training demonstrated greater improvements than those who were trained in memory and reasoning domains [12], with gains durable for 10 years [13], including a 29% reduction in dementia risk [14].

SOP training may be particularly well-suited for aging PWH. First, SOP deficits in PWH are well documented [3, 15–18] with SOP being among the cognitive domains demonstrating the greatest decline from early to late stages of HIV for all ages [19–23]. Second, evidence suggests SOP is the cognitive domain that may be most improved by cognitive training [8]. Third, according to Salthouse’s processing-speed theory [24] (orginally proposed by Birren, 1965 [25]), SOP slows with age [26], and such declines can occur at all stages of processing, from the speed at which information is encoded to the execution of a response [27, 28]. This reduction in SOP places demands on other cognitive systems such that age-related decrements in memory, reasoning, and fluency may be mediated through differences in SOP [29]. In fact, a study of 186 PWH found that SOP “fully mediated the effects of age on learning, memory, and executive functioning and partially mediated the effect of major depressive disorder on learning and memory” [30](p. 806) while other HIV studies show SOP deficits impair real-world functioning [2, 31].

To date, few studies have examined SOP training among aging PWH. In one of the first studies of its kind, 46 PWH 40 and older were randomized into either: (1) a no-contact control group, or (2) a 10-h SOP training group [32]. Compared to the control group immediately after training completion, those in the SOP condition improved on the Useful Field of View (UFOV®) test, a cognitive test of visual SOP and attention, as well as on a performance-based measure of everyday functioning. In a subsequent study of 88 PWH with HAND, an individualized cognitive training approach was employed that targeted an individual’s specific impaired cognitive domains, with a priority for SOP impairments. This study found that participants with SOP training in their individualized program experienced the greatest cognitive performance gains overall, which were greatest for the SOP domain [11]. In the current Think Fast Study, we sought to improve upon the prior cognitive training studies in PWH by: (1) including longitudinal follow-up, (2) including a larger sample size, and (3) comparing dosage effects of 10 vs 20 h of SOP training. There were several aims of the Think Fast Study. Overall, the aims focused on the impact of speed of processing training on UFOV® performance (Aim 1A), on other cognitive outcomes (Aim 1B; [33]), on everyday functioning (Aim 2; [34]), and on quality of life (Aim 3; [35]). Thus, the purpose of this article was to examine Aim 1A, to determine whether 10 vs 20 h of SOP training would improve UFOV® performance at post-test immediately after training, and at year 1 and year 2 follow up compared to those in an active, contact control condition.

## Methods

### Participants and Procedure

In the Think Fast Study, a 3-group 2-year longitudinal efficacy study, 216 PWH with HAND or borderline HAND (see HAND diagnosis below) were randomized to either: (1) 10 h of SOP training (*n* = 70); (2) 20 h of SOP training (*n* = 73), or (3) 10 h of contact control training (*n* = 73; i.e., computer/social engagement control). Participants were administered a 3-to-4-h assessment at baseline, posttest immediately after training, year 1, and year 2. These assessments repeated the measures at baseline and included everyday functioning, psychosocial (i.e., depressive symptomatology), and substance use measures. It also included a full cognitive battery, which also included UFOV® [36]. Analyses examined the effects of SOP training over time on UFOV® outcomes, and whether these changes differed by dosage.

**Fig. 1:**
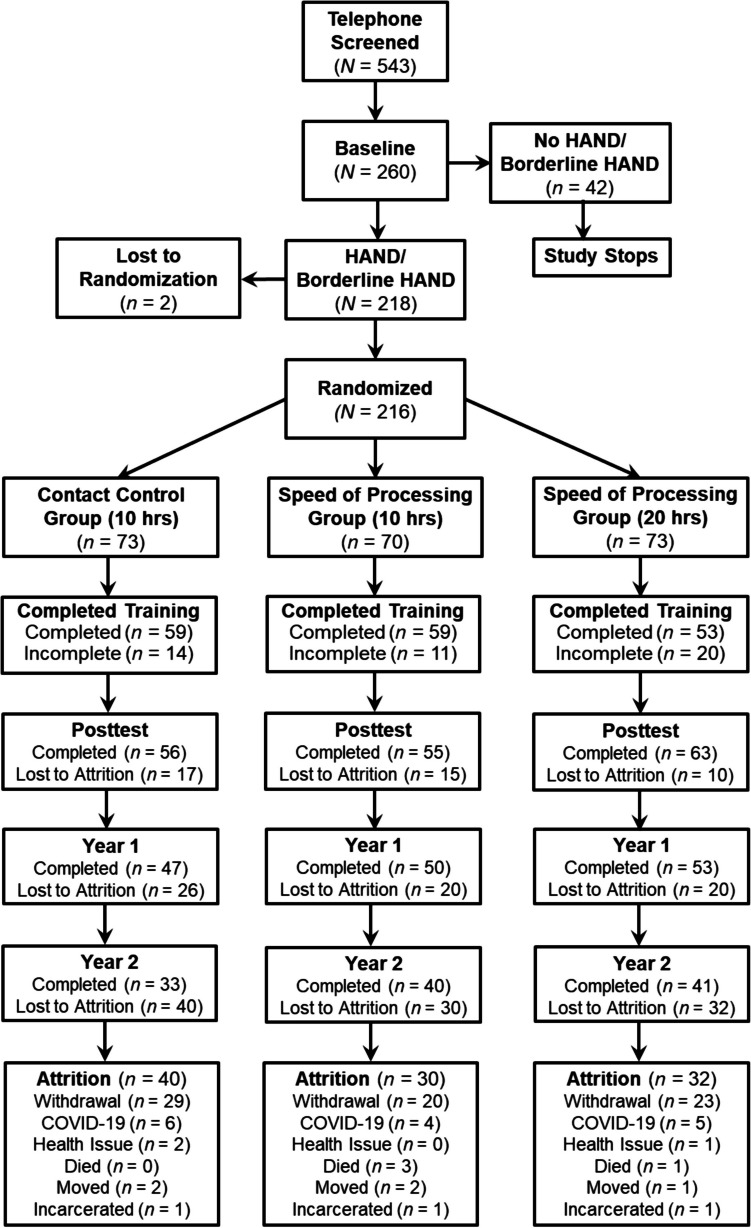
*THINK FAST consort diagram of* a randomized controlled trial of speed of processing training dosages compared to a contact control group

Recruitment materials were placed in a university HIV/AIDS clinic in an urban area in Birmingham, Alabama. These materials directed participants to call the research office, at which point a telephone screening was used to discern eligibility. Participants were eligible if they were 40 years old or older, diagnosed with HIV for at least 1 year, and were English speaking. Significant neuromedical comorbidities (e.g., schizophrenia) were exclusionary factors, as well as any other conditions (e.g., legally deaf or blind, history of brain trauma) that could impact feasibility and ability to participate in study procedures (e.g., cognitive assessment, SOP training) [3]. If eligible, participants were administered a baseline assessment to determine final eligibility based on presenting with HAND (see HAND diagnosis below). This study was approved by a human subject ethics board (protocol #: F160122002); participants provided written consent using a form approved by the University of Alabama at Birmingham IRB. The data collection period for this study commenced in September 2016 and concluded in March 2020. However, the study was cut short due to the COVID-19 pandemic, leading to the inability to collect year 2 follow-up data from 15 participants. Thus, all the data obtained in the study represent the pre-COVID-19 period. The ClinicalTrials.gov number of this study is NCT02758093. A consort diagram can be found in Fig. 1.

### Measures

#### Demographic Information

Participants were administered a pencil-and-paper questionnaire which collected demographic information such as age, race (non-white = 0; white = 1), gender (female = 0; male = 1), education, and yearly household income. Education was measured in years of education attained, ranging from 1 to 20 years. Yearly household income was measured in 10,000 dollar increments, ranging from 0 to above 100,000 dollars (USD).

#### HIV Characteristics

The university HIV/AIDS clinic where recruitment occurred provided health information related to participants’ HIV status. This information included current viral load, current CD4 + lymphocyte count, and nadir CD4 + lymphocyte count (nadir is the lowest point over time, which is significant as this shows the lowest point in which the immune system has been compromised by HIV which could have physiological and neurological consequences over time).

#### Depression

Depression screening was included to further describe sample comorbidities. Depressive symptoms were captured using the Center for Epidemiologic Studies Depression Scale-Revised (CES-Depression) [37] with scores ranging from 0 to 60; a score of 16 or higher indicates significant depressive symptomology.

#### Substance Use

Participants were asked about current tobacco use (0 = *no*; 1 = *yes*), number of cigarettes smoked/day, frequency of alcohol use (0 = *never*; 5 = *4–7 times/week*), and number of drinks on days drinking (1 = *not applicable/don’t drink*, 2 = *1–2 drinks*,…6 = *10 or more drinks*). A urine toxicology screen tested for marijuana, amphetamine, methamphetamine, cocaine, and opioids. Participants were marked as positive (i.e., UTOX +) if the presence of these substances were observed. Participants were not excluded from the study or analyses if they were positive on the UTOX + or indicated high levels of alcohol or tobacco use. To capitalize on the ecological validity of the study that reflects the larger HIV population in terms of substance use, all participants regardless of substance use status were included in the analyses.

#### HAND Diagnosis

At baseline, a comprehensive seven domain cognitive battery was administered to determine HAND status. The standardized normed-based cognitive tests for each domain included: speed of information processing (Trail Making Test A, Digit Symbol Task, Symbol Search), attention/working memory (Letter Number Sequencing, Paced Auditory Serial Addition Test), executive functioning (Wisconsin Card Sorting Test, Trail Making Test B), learning and delayed recall [(2 domains), Hopkins Verbal Learning Test, Brief Visuospatial Memory Test)], verbal fluency (Controlled Oral Word Association Test and Animal and Action), and motor skills (Grooved Pegboard Test dominant and nondominant hands). Raw scores from each cognitive measure in the battery (see protocol paper for full battery [36]) were converted to demographically corrected *T* scores that accounted for age, gender, education, and race when available, which were then used to generate clinical ratings based on published approaches, with global clinical ratings of 5 or greater (indicating impairment in two or more cognitive domains) reflecting HAND [38–40]. For the current study, participants with borderline HAND (i.e., clinical ratings of 4 or greater) were included to allow for a generalizable sample of PWH with a range of functioning and who may be at risk for future decline [41].

#### Speed of Processing

We used the UFOV® test [42], one of the most extensively used SOP measures in aging research [43]. Prior work demonstrates the utility of the UFOV® test as a clinically valid measure of this domain [44] and its response to SOP training [12]. The UFOV® test was administered via a touch screen computer with participants wearing vision correction if needed. The UFOV® test includes four subtests which reflect presentation time in milliseconds at which stimuli can be detected better than chance (at 75% accuracy) across varying conditions. Task 1 (UFOV1) measures SOP, and involves the stimuli being presented centrally with no other targets. Task 2 (UFOV2) measures divided attention and involves a pair of stimuli with one presented centrally and one peripherally. Task 3 (UFOV3) measures selective attention and involves the same protocol as task 2 together with irrelevant distracters. Task 4 (UFOV4) measures selective attention under more demanding conditions; it involves the same protocol as task 3 except the center task is more demanding in which the participants must determine whether the two objects in the center box are the same or different. The UFOV® test includes scores for the four tasks and a total sum across all four tasks; higher scores reflect worse SOP and visual attention. As UFOV® was originally developed to be a validated proxy measure of hazardous driving in older adults using tasks 1–3, risk categories were developed based on the UFOV® driving literature [43, 45]. Thus, the UFOV® test scores can also be used to generate a five-category UFOV® risk index (1 = *very low risk*, 2 = *low risk*, 3 = *low–moderate risk*, 4 = *moderate–high risk*, and 5 = *high risk*). Subgroup analyses in the current study examined results among participants with baseline UFOV® risk categories greater than 1.

### SOP Training Groups and Contact Control Group Training Protocols

Soon after baseline, if participants had HAND or borderline HAND, they were then randomized to either: (1) 10 h of SOP training (*n* = 70); (2) 20 h of SOP training (*n* = 73), or (3) 10 h of Internet navigation training (*n* = 73; i.e., contact control group). Our randomization strategy used stratification to ensure a balanced assignment between women/men, African Americans/Caucasians, and UFOV® risk (RISCAT = 1 vs 2–5). Within each combination of strata, a randomization list was generated utilizing a permuted block approach with block sizes of 3 and 6. Participants did not have to have a particular RISCAT to be assigned to a particular group; this was to ensure that no particular group had more individuals with UFOV® impairment than another. Study staff did not determine the randomization; the principal investigator in consultation with the study statistician developed the randomization key. As participants entered the study, the principal investigator used each participant’s characteristics (i.e., RISCAT score) to select the preselected group assignment; the principal investigator did not have direct contact with the participants. Participants in all three groups completed their assigned intervention in a computer lab in our research center, under the supervision of trained research staff who monitored participants’ progress, assisted with logging onto the computer program, and maintained treatment fidelity. Breaks were provided as needed.

Participants in the SOP training groups completed 10 and 20 h respectively of SOP cognitive training modules which were accessible at BrainHQ (www.brainhq.com). These modules chosen for training included Master Gardner, Bird Safari, Jewel Diver, Sweep Seeker, and Road Tour. These SOP exercises were designed to improve visual attention and visual speed of processing as various visual targets (i.e., birds, road signs, bubbles, jewels) were scattered centrally and peripherally on the screen requiring participants to visually track the movement or the location of quickly presented targets. The intent was that these exercises would help expand one’s useful field of view by requiring participants to broaden their visual field beyond focusing on central target alone. These exercises were somewhat similar at times, but deliberately not identical, to the UFOV® test. Manipulations used to increase difficulty included decreasing the duration for which the visual stimuli are presented, adding visual distractors, increasing similarity between targets/distracters, and presenting visual targets over a broader spatial expanse, which expands one’s useful field of view. Further, auditory sounds were also present that reinforced correct responses (i.e., a positive ping sound) or indicated incorrect responses (i.e., a negative clunk sound) in real time. An algorithm was used to present these stimuli in various milliseconds that sped up or slowed down in response to how fast participants correctly responded. Conceptually, this fosters a challenging cognitive environment that encourages positive neuroplasticity [46].

Participants in the contact control group completed 10 h of engaging in modules in which they used the Internet to find health information, identify geographic information (i.e., states), and other web-based activities. This contact control intervention was developed by the research team to be engaging without having any obvious cognitive challenge that could improve cognitive performance. This contact control intervention was developed to control for computer contact time and staff contact time.

Staff kept track of the number of hours each participant engaged in the training activities, and the online program also monitored the amount of time participants engaged in each exercise. The correlation between our reports and the software on time engaged in cognitive training was very high (*r* = 0.91; *p* = 0.001). Given transportation difficulties experience by many participants [47], to cut down on the number of trips to the research center they had the option to engage in up to two hours of training at a time, usually spread out over a 12-to-20-week period. Regarding study activities, the average percentage of assigned training activity hours (either SOP or contact control) as logged by study staff was high at 87.92% (*SD* = 30.59%). The average number of SOP computer training hours reported by the software was 6.94 (*SD* = 2.97) for the 10-h group, and 13.88 (*SD* = 4.99) for the 20-h group; the average number of SOP hours engaged in training was 8.88 (*SD* = 2.96) hours and 18.25 (*SD* = 5.22) hours for the 10-h group and 20-h group, respectively. Once the training schedule was completed, the participants were administered the posttest assessment; participants did not have continued access to training after posttest. At posttest, participants were also administered a survey of acceptability of the training, including an item asking how enjoyable the computer games were (1 = *not all*, 2 = *a little*, 3 = *moderately*, 4 = *very much*, 5 = *extremely*).

### Data Analysis

Analysis began by examining balance between study groups with respect to participant characteristics (e.g., age, gender, race, education, HIV markers, etc.), baseline outcomes, and participation in follow-up data collection, using measures of effect size, such as the proportion of variance explained, *R*^*2*^ (small ~ 0.02, medium ~ 0.13, large ~ 0.26) and Cramer’s *V* (small ~ 0.07, medium ~ 0.21, large ~ 0.35), for cross-tabulations comparing three groups [48]. Then, participants’ characteristics and baseline outcomes were examined for association with study attrition (i.e., whether participants dropped out of the study or not) using measures of effect size, such as Cohen’s *d* (small ~ 0.2, medium ~ 0.5, large ~ 0.8) and Cramer’s *V* (small ~ 0.1, medium ~ 0.3, large ~ 0.5) for cross-tabulations comparing two groups [48]. Characteristics with non-trivial magnitude of association (l*d*l ≥ 0.25) with attrition were used as controlling covariates in subsequent analysis steps to reduce potential bias caused by missing data [49]. Next, intention-to-treat-analyses for UFOV® total as the main outcome as well as for each of the UFOV® test scores, linear mixed-effect models with subject-specific coefficients were fitted to estimate between-group mean differences at the follow-up time-points. Although the UFOV® tests are successive, they each provide a nuanced look at useful field of view performance as they increase in complexity and difficulty; thus, they were examined separately in these analyses. Model fixed effects included: baseline values, indicator variables for time, group, time-by-group interaction, and adjusting covariates associated with missing data. These analytic models used all available data at each time-point and model covariates were determined by evaluating effect size (l*d*l ≥ 0.25) of sample characteristics in relation to attrition. Baseline data were used to estimate a pooled standard deviation for each outcome score, which was then used to standardize the between-group contrasts and provide a measure of effect size (Cohen’s *d*). The Satterthwaite approximation to degrees of freedom was used for significance tests of between-group differences in outcomes for all three groups. A multiple inference correction was applied to the tests of between-group comparisons in outcomes using a False Discovery Rate (FDR) approach with a target of 10% FDR level [50]. A subgroup analysis including participants with baseline UFOV® risk categories greater than 1 was conducted following similar steps as described above. The enjoyability of the computer games was analyzed with a general linear model with robust standard errors. Analyses were conducted using R software version 4.1.1 [51]. Data and data analyses are available upon request.

## Results

### Participant Characteristics

Table 1 shows baseline characteristics of the *N* = 216 participants in the study. Measures of effect size did not suggest any major imbalance among the study groups.

**Table 1:** Baseline demographic and clinical characteristics of 216 participants with HIV in a randomized controlled trial of speed of processing training dosages compared to a contact control group

Variable	Contact control group (*n* = 73)		10-h cognitive training (*n* = 70)		20-h cognitive training (*n* = 73)		Effect size
	*n* (%)	Mean (*SD*)	*n* (%)	Mean (*SD*)	*n* (%)	Mean (*SD*)	
Age		50.16 (6.68)		51.36 (6.34)		51.52 (6.55)	R2 = 0.01
Gender							V = 0.06
Female	30 (41.10%)		27 (38.57%)		25 (34.25%)		
Male	43 (58.90%)		43 (61.43%)		48 (65.75%)		
Race/ethnicity							V = 0.03
Non-white	60 (82.19%)		59 (84.29%)		60 (82.19%)		
White	13 (17.81%)		11 (15.71%)		13 (17.81%)		
Education (years)		12.30 (2.49)		12.59 (2.37)		12.33 (1.92)	R2 = 0
Household income ($10 K)		1.89 (1.60)		1.77 (1.42)		1.84 (1.44)	R2 = 0
Years diagnosed with HIV		15.69 (8.93)		14.19 (8.14)		18.18 (8.03)	R2 = 0.04
Current CD4 + T lymphocyte count/mm^3^		685.67 (414.91)		693.90 (360.54)		562.00 (335.74)	R2 = 0.03
Nadir CD4 + T lymphocyte count/mm^3^		289.74 (275.60)		269.77 (263.95)		252.54 (288.83)	R2 = 0
No. of prescribed medications		6.47 (4.05)		6.27 (4.00)		7.53 (5.64)	R2 = 0.01
Prescribed ART							V = 0.07
No	4 (5.48%)		2 (2.86%)		3 (4.11%)		
Yes	65 (89.04%)		61 (87.14%)		66 (90.41%)		
Unknown	4 (5.48%)		7 (10.00%)		4 (5.48%)		
CES-depression		19.95 (11.60)		18.03 (11.36)		17.16 (10.23)	R2 = 0.01
Locus of control		26.84 (6.24)		27.73 (5.94)		28.49 (6.21)	R2 = 0.01
Alcohol use frequency							V = 0.1
Never	31 (42.47%)		32 (45.71%)		29 (40.28%)		
Monthly or less	15 (20.55%)		17 (24.29%)		15 (20.83%)		
Two to four times a month	14 (19.18%)		6 (8.57%)		15 (20.83%)		
Twice weekly or more	13 (17.81%)		15 (21.43%)		13 (18.06%)		
No. of drinks on a drinking day		3.06 (1.97)		2.75 (2.27)		2.77 (1.90)	R2 = 0
Currently using tobacco	42 (57.53%)		40 (57.14%)		37 (50.68%)		V = 0.06
Cigarettes per day		11.76 (7.62)		8.15 (5.98)		10.05 (7.28)	R2 = 0.04
UTOX +	30 (42.25%)		28 (40.58%)		35 (50.00%)		V = 0.06
Study activities							
Hours logged in training activities		8.38 (3.52)		8.88 (2.96)		18.25 (5.22)	R2 = 0.56
% of prescribed training activity hours logged		83.84 (35.22)		88.81 (29.57)		91.25 (26.11)	R2 = 0.01
Hours of computerized cognitive training		NA (NA)		6.94 (2.97)		13.88 (4.99)	R2 = 0.42
Cognitive function							
Global function T		43.59 (5.55)		42.75 (5.29)		43.13 (4.40)	R2 = 0
Global clinical rating Scale		5.16 (1.18)		5.20 (1.14)		5.29 (0.96)	R2 = 0
HAND							V = 0.13
Impaired	45 (61.64%)		50 (71.43%)		55 (75.34%)		
Normal	28 (38.36%)		20 (28.57%)		18 (24.66%)		
Baseline outcomes							
UFOVTOTAL (ms)		732.26 (312.77)		778.89 (305.93)		758.15 (344.47)	R2 = 0
UFOV1 (ms)		35.25 (47.62)		42.50 (66.20)		44.90 (61.95)	R2 = 0
UFOV2 (ms)		108.89 (100.72)		111.56 (107.84)		117.67 (116.06)	R2 = 0
UFOV3 (ms)		213.51 (120.71)		224.39 (114.29)		210.70 (121.99)	R2 = 0
UFOV4 (ms)		374.62 (112.51)		400.44 (99.70)		384.88 (109.60)	R2 = 0.01
RISCAT		1.75 (0.91)		1.81 (1.04)		1.89 (1.15)	R2 = 0
Follow-up data collection							V = 0.02
Post	55 (75.34%)		55 (78.57%)		63 (86.3%)		
Year 1	47 (64.38%)		50 (71.43%)		53 (72.6%)		
Year 2	33 (45.21%)		40 (57.14%)		41 (56.16%)		

*R*^2^ R-squared, *V* Cramer’s V, *ART* antiretroviral therapy, *CES*-Depression Center for Epidemiological Studies Depression Scale, *ms* milliseconds in which correctly responded, *no*. number, *RISCAT* UFOV risk category, *SD* standard deviation, *UFOV* Useful Field of View, *UTOX* + urine toxicology screen indicating a positive result, $10 K = ten thousand dollars

R2: ~ 0.02 small, ~ 0.13 medium, ~ 0.26 large. In this table, Cramer’s *V*: ~ 0.07 small, ~ 0.21 medium, ~ 0.35 large. In the *n* = 73 in the Control group, of the 60 non-white people, 59 (98%) were African American and 1 person reported other (2%). In the *n* = 70 in the 10 h of SOP group, of the 59 non-white people, 57 (96%) were African American and 1 was Hispanic (2%) and 1 person reported other (2%). Of the *n* = 73 in the 20 h of 2OP group, of the 60 non-white people, 56 (94%) were African American, 2 were Hispanic (3%) and 2 people reported other (3%)

On average, the participants in this study were 51.01 years old (*SD* = 6.53). Approximately two-thirds of the participants were male and the majority identified as Black, Indigenous, and People of Color (BIPOC). The participants had an average of 12.4 years of education (*SD* = 2.27) and reported an average income of approximately $18,300 (*SD* = $14,800). Additionally, there were 93 participants (44.29%) with positive urine toxicology (UTOX +). Participants’ CES-Depression scores had an average score of 18.38 (*SD* = 11.08), with *n* = 112 (51.85%) reporting CES-Depression ≥ 16, suggesting relevant depressive symptomatology.

As a whole, these participants were medically stable with HIV. On average participants have been living with HIV for 16.05 years (*SD* = 8.50), had current CD4 + T lymphocyte count/mm^3^ of 649.61 (*SD* = 375.75), nadir CD4 + T lymphocyte count/mm^3^ of 270.14 (*SD* = 275.80), and the majority were on antiretroviral therapy. Overall, the Global Clinical Rating Scale was 5.22 (*SD* = 1.09) and the percentage of HAND Impaired was 69.44% (*n* = 150).

### Attrition

The bottom of Table 1 shows frequencies of participation at each follow-up data collection time-point. Overall, *n* = 173 (80.09%), *n* = 150 (69.44%), and *n* = 114 (52.78%) participants were retained at the post, year 1, and year 2 time-points, respectively. Supplemental Table 1 shows comparisons of baseline characteristics between the *n* = 114 participants who remained in the study with follow-up data for all the visits vs. the *n* = 102 who dropped out of the study and did not have data for all follow-up time-points. We defined those with all the follow-up data as those who remained in the study and were administered the baseline, posttest, year 1, and year 2 assessments. As per the measures of effect size, there was no single characteristic strongly associated with attrition, but moderate differences (*d* ≥ 0.25) were observed in nadir CD4 + and number of prescriptions. These two characteristics were used as covariates in outcome analyses.

### Completion of Training

As seen in Table 1 and reported earlier in the description of the training protocols, there were no differences in the amount of training completed between the groups. Yet, there was concern given the high levels of depressive symptomatology in the sample that this could have influence completion of training. Thus, an analysis was conducted examining if there was a relationship between depression scores and hours of training completed. It was not feasible to compare the hours directly because participants where prescribed different hours of activity (either 10 or 20), but we did compare the percentage of activity hours they completed. A Pearson correlation and *t*-test between CES-Depression and percentage of prescribed hours completed revealed no relationship (*r* = 0.00062, 95% CI = − 0.134, 0.135; *t*(*df* = 210) = 0.009, *p* = 0.993). The estimate of correlation is almost at zero and the *t*-test does not support a relationship between percentage of training completion and CES-Depression.

### Effects of Training

Table 2 presents model-estimated outcome means and between-group contrasts at the follow-up time-points. All models included baseline outcome values as an adjusting covariate. The multiple inference adjustment indicated that *P* values ≤ 0.02 were considered statistically significant at a 10% False-Discovery-Rate (FDR) level. (This *P* value threshold of 0.02 is not set a priori, it is instead a result. It results from applying the FDR correction to the 54-contrast *p* values presented in Table 3 at 10% FDR level. In presentation, this would be equivalent to applying a threshold of 0.1 to the multiplicity-adjusted *p* values (referred to as *q*-values in some disciplines). We opted to leave the raw *p* values for readers who might want to replicate the correction or use the raw *p* values in any other way). Non-linear trajectories over time were observed for all outcomes, with larger effects, relative to the control group, at the post-intervention and year 2 time-points, compared to the effects at year 1. As an expected result from dropout, effect estimates had a much higher degree of uncertainty at the year 2 time-point, as quantified by standard errors and confidence intervals. At the post-intervention time-point, small beneficial SOP training effects were observed for the 10-h group in UFOVTOTAL (*d* = 0.28, *p* = 0.002), UFOV2 (*d* = 0.3, *p* = 0.013), UFOV3 (*d* = 0.27, *p* = 0.014), and RISCAT (*d* = 0.29, *p* = 0.026), albeit the effect on RISCAT did not cross the significance threshold. Effects were of larger magnitude and statistically significant for the 20-h group in these same outcomes, i.e., UFOVTOTAL: *d* = 0.43, *p* < 0.001; UFOV2: *d* = 0.5, *p* < 0.001; UFOV3: *d* = 0.42, *p* < 0.001; and RISCAT: *d* = 0.49, *p* = 0.001. These results indicated higher benefit with more training; however, doubling the dose did not result in doubling the point estimates of effect.

**Table 2:** A randomized controlled trial of 216 participants with HIV assigned to speed of processing training dosages or a contact control group comparing UFOV® treatment outcomes by group at posttest, year 1, and year 2 follow-up

Outcomes by group and between-group contrasts	POST			Year 1			Year 2		
	Mean (SE)	*P*	*d* (95% CI)	Mean (SE)	*P*	*d* (95% CI)	Mean (SE)	*P*	*d* (95% CI)
UFOVTOTAL (ms)									
Control	699.93 (20.63)	–	–	680.92 (27.38)	–	–	689.29 (46.71)	–	–
10 h	608.47 (20.44)	–	–	672.51 (26.57)	–	–	617.16 (41.99)	–	–
20 h	560.81 (19.12)	–	–	627.86 (25.85)	–	–	587.23 (41.32)	–	–
Control vs. 10 h	91.46 (29.04)	0.002	0.28 (0.1, 0.46)	8.41 (38.16)	0.826	0.03 (− 0.21, 0.26)	72.13 (62.81)	0.253	0.22 (− 0.16, 0.61)
Control vs. 20 h	139.12 (28.17)	< 0.001	0.43 (0.26, 0.61)	53.06 (37.7)	0.16	0.16 (− 0.07, 0.4)	102.07 (62.39)	0.104	0.32 (− 0.07, 0.7)
10 h vs. 20 h	47.66 (28.02)	0.09	0.15 (− 0.02, 0.32)	44.65 (37.1)	0.23	0.14 (− 0.09, 0.37)	29.94 (58.93)	0.612	0.09 (− 0.27, 0.46)
UFOV1 (ms)									
Control	30.8 (4.46)	–	–	32.2 (4.78)	–	–	34.76 (5.9)	–	–
10 h	19.35 (4.46)	–	–	35.05 (4.64)	–	–	24.61 (5.19)	–	–
20 h	20.07 (4.14)	–	–	19.25 (4.52)	–	–	16.53 (5.14)	–	–
Control vs. 10 h	11.44 (6.31)	0.07	0.19 (− 0.02, 0.4)	− 2.85 (6.66)	0.669	− 0.05 (− 0.27, 0.17)	10.15 (7.87)	0.197	0.17 (− 0.09, 0.43)
Control vs. 20 h	10.73 (6.09)	0.079	0.18 (− 0.02, 0.39)	12.95 (6.59)	0.05	0.22 (0, 0.44)	18.24 (7.83)	0.02	0.31 (0.05, 0.57)
10 h vs. 20 h	− 0.72 (6.08)	0.906	− 0.01 (− 0.21, 0.19)	15.8 (6.48)	0.015	0.27 (0.05, 0.48)	8.08 (7.31)	0.269	0.14 (− 0.11, 0.38)
UFOV2 (ms)									
Control	103.5 (9.23)	–	–	92.97 (9.89)	–	–	87.43 (12.14)	–	–
10 h	70.87 (9.22)	–	–	73.95 (9.59)	–	–	60.41 (10.7)	–	–
20 h	48.9 (8.56)	–	–	83.22 (9.34)	–	–	53.2 (10.59)	–	–
Control vs. 10 h	32.62 (13.05)	0.013	0.3 (0.06, 0.54)	19.02 (13.77)	0.168	0.18 (− 0.07, 0.43)	27.02 (16.2)	0.096	0.25 (− 0.05, 0.54)
Control vs. 20 h	54.6 (12.61)	< 0.001	0.5 (0.27, 0.74)	9.75 (13.63)	0.474	0.09 (− 0.16, 0.34)	34.23 (16.13)	0.034	0.32 (0.02, 0.61)
10 h vs. 20 h	21.98 (12.6)	0.082	0.2 (− 0.03, 0.43)	− 9.27 (13.41)	0.49	− 0.09 (− 0.33, 0.16)	7.21 (15.06)	0.632	0.07 (− 0.21, 0.34)
UFOV3 (ms)									
Control	205.39 (9.13)	–	–	187.58 (11.28)	–	–	202.92 (18.01)	–	–
10 h	173.56 (9.05)	–	–	182.26 (10.94)	–	–	163.9 (16.08)	–	–
20 h	154.79 (8.47)	–	–	166.5 (10.65)	–	–	157.39 (15.85)	–	–
Control vs. 10 h	31.82 (12.86)	0.014	0.27 (0.05, 0.48)	5.32 (15.71)	0.735	0.04 (− 0.21, 0.3)	39.02 (24.15)	0.108	0.33 (− 0.07, 0.73)
Control vs. 20 h	50.6 (12.47)	< 0.001	0.42 (0.22, 0.63)	21.08 (15.54)	0.175	0.18 (− 0.08, 0.43)	45.53 (24.01)	0.06	0.38 (− 0.02, 0.78)
10 h vs. 20 h	18.77 (12.41)	0.131	0.16 (− 0.05, 0.36)	15.76 (15.29)	0.303	0.13 (− 0.12, 0.38)	6.52 (22.59)	0.773	0.05 (− 0.32, 0.43)
UFOV4 (ms)									
Control	358.7 (9.53)	–	–	366.78 (12.4)	–	–	364.97 (20.7)	–	–
10 h	348.61 (9.45)	–	–	382.39 (12.04)	–	–	373.63 (18.65)	–	–
20 h	339.4 (8.84)	–	–	359.68 (11.7)	–	–	353.19 (18.33)	–	–
Control vs. 10 h	10.09 (13.43)	0.453	0.09 (− 0.15, 0.34)	− 15.6 (17.29)	0.367	− 0.15 (− 0.46, 0.17)	− 8.65 (27.87)	0.757	− 0.08 (− 0.59, 0.43)
Control vs. 20 h	19.3 (13.02)	0.139	0.18 (− 0.06, 0.42)	7.11 (17.07)	0.677	0.07 (− 0.25, 0.38)	11.78 (27.66)	0.671	0.11 (− 0.4, 0.62)
10 h vs. 20 h	9.21 (12.96)	0.478	0.09 (− 0.15, 0.32)	22.71 (16.81)	0.178	0.21 (− 0.1, 0.52)	20.43 (26.16)	0.436	0.19 (− 0.29, 0.67)
RISCAT									
Control	1.73 (0.1)	–	–	1.66 (0.1)	–	–	1.61 (0.13)	–	–
10 h	1.43 (0.1)	–	–	1.55 (0.1)	–	–	1.33 (0.11)	–	–
20 h	1.29 (0.09)	–	–	1.47 (0.1)	–	–	1.18 (0.11)	–	–
Control vs. 10 h	0.3 (0.13)	0.026	0.29 (0.03, 0.55)	0.11 (0.14)	0.455	0.1 (− 0.17, 0.37)	0.28 (0.17)	0.094	0.27 (− 0.05, 0.59)
Control vs. 20 h	0.44 (0.13)	0.001	0.43 (0.18, 0.68)	0.19 (0.14)	0.171	0.19 (− 0.08, 0.45)	0.43 (0.17)	0.009	0.42 (0.1, 0.73)
10 h vs. 20 h	0.14 (0.13)	0.277	0.14 (− 0.11, 0.38)	0.09 (0.14)	0.532	0.08 (− 0.18, 0.35)	0.15 (0.16)	0.325	0.15 (− 0.15, 0.44)

*ms* milliseconds in which correctly responded, *RISCAT* UFOV risk category, *UFOV* Useful Field of View

Estimates from longitudinal models adjusted for baseline outcome, number of prescriptions, and Nadir CD4

Cohen’s *d*: ~ 0.2 small, ~ 0.5 medium, ~ 0.8 large. *P*-values ≤ 0.02 are considered statistically significant at a 10% False-Discovery-Rate level

At the year 1 time-point, training effects were also in the direction of benefit to participants for all outcomes; however, the sizes of the effects were reduced to trivial-to-small magnitude for both the 10-h and 20-h training groups and were not statistically significant. At year 2, beneficial effects of small magnitude were observed again in the 10-h group, with UFOVTOTAL: *d* = 0.22, *p* = 0.253; UFOV2: *d* = 0.25, *p* = 0.096; UFOV3: *d* = 0.33, *p* = 0.108; and RISCAT: *d* = 0.27, *p* = 0.094, and were not statistically significant. Similarly, effects were again of larger magnitude in the 20-h group, with UFOVTOTAL: *d* = 0.32, *p* = 0.104; UFOV1: *d* = 0.31, *p* = 0.02; UFOV2: *d* = 0.32, *p* = 0.034; UFOV3: *d* = 0.38, *p* = 0.06; and RISCAT: *d* = 0.42, *p* = 0.009, with the effects on UFOV1 and RISCAT crossing the significance threshold.

#### Subgroup Analysis

Participants already performing well in UFOV® may not have had room to improve from training and as such, may have reduced the training effect. To explore this further, participants with a RISCAT < 1 were removed in a subgroup analysis. Supplemental Table S2 shows characteristics of the *n* = 108 participants included in the subgroup analysis of participants with baseline RISCAT ≥ 2. Supplemental Table S3 shows modeling results for the study outcomes in the subgroup. Overall, training effects on all outcomes were of larger magnitude compared to the main analysis. Similar to the main analysis, effects were larger at post-intervention, compared to the year 1 and year 2 time-points; however, there was less variation between effects in year 1 and year 2.

#### Exploratory Analysis

Since substance use has been shown to impact cognition, it could also impact cognitive training. In an exploratory analysis, we fitted a model for UFOVTOTAL with a 3-way interaction for time by group by UTOX + , resulting in an *F*-test statistic of *F*(6, 375.54) = 0.3333, *p* = 0.9193, suggesting no support in this case for UTOX as a moderator. As a comparison, the *F* test for the intervention effect on UFOVTOTAL (a 2-way interaction test of time by group) resulted in *F*(6, 392.98) = 2.861, *p* = 0.009, and was significant. This suggests that substance use did not impact the training effect observed in this study.”

#### Enjoyability of Computer Training

At posttest, as a measure of palatability and acceptance of the intervention, we asked participants how much they enjoyed the computer games. These games referred to the computer activities involved across the treatment arms as both the control group and the SOP groups engaged in various interactive computer tasks. A total of *n* = 138 participants provided ratings regarding the enjoyability of the computer games, with an overall average enjoyment of *M*(*SD*) = 3.7(1.1), a rating between *moderately* = 3 and *very much* = 4. Average enjoyment was slightly higher in the control group (*M*(*SD*) = 3.9(0.9), *n* = 41), compared to the 10-h (*M*(*SD*) = 3.6(1.1), *n* = 49) and 20-h (*M*(*SD*) = 3.6(1.2), *n* = 48) groups. These differences represented a small effect size: *R*^2^ = 0.018, Adjusted-*R*^2^ < 0.01, *F*(2,135) = 1.49, *p* = 0.23.

## Discussion

In the Think Fast Study, we confirmed that a larger dose of SOP training resulted in a larger therapeutic benefit in SOP performance; however, the double dose does not translate to a doubled therapeutic effect. In a systematic review of 52 cognitive training studies in community-dwelling older adults, Lampit et al. [8] concluded there exists a U-shaped dosage-therapeutic response to training as well, with training beyond 20 h also resulting in diminished therapeutic benefit. Overall, our results reflect small to medium effects of training on SOP performance.

Concerning durability of treatment effect, it was somewhat maintained over a two-year period, especially with more training, as expected. The training effect did seem to decrease in year 1 and improve in year 2; this could be just random variation, or it could indicate a latent effect of training that emerges over time. In both the ACTIVE [12] and Accelerate Study [52] which both used SOP training in community-dwelling older adults and monitored the effect over 2 or more years, both found that the training effect dissipated slowly over time. Yet in ACTIVE, booster training helped maintain the training effect. Our posthoc analyses showed that training effects were larger for those participants with worse SOP at baseline, which suggests who may benefit the most from SOP training. Yet, our study still showed that effects atrophy over time from initial training which is reflective of the wider cognitive training literature [8, 12, 52]. As these cognitive skills are no longer reinforced behaviorally through repeated training, they weakened through disuse. Thus, booster training may be a way to maintain such cognitive gains over time.

Based on the ACTIVE Study, the NINR/NIA announced that SOP training can help enable “older people to maintain their cognitive abilities as they age,” even 10 years after training [13]. More recently, in an examination of the ACTIVE data over 10 years, Edwards et al. found that compared to no training, memory training, and reasoning training, SOP training significantly reduced the risk of dementia (i.e., 10% lower hazard of dementia observed with each additional SOP training session) [14]. These encouraging findings are supported by cortical electrophysiological studies that indicate that adults who receive SOP training, compared to controls, experience increased N2pc and P3b amplitudes reflective of capacity enhancement and attentional allocation [53].

### Implications for Practice

Regrettably, few behavioral interventions have tried to improve cognition in this pharmacologically-burdened population [54], and pharmacological cognitive interventions produce adverse side effects in a population already experiencing multiple comorbidities [52, 54–57]. PWH also suffer from higher rates of depression, poorer self-rated health and health-related quality of life, loss of control, and poorer everyday functioning compared to the seronegative population [36, 58]. Fortunately, prior studies targeting SOP deficits (e.g., ACTIVE) show that SOP training effects transferred to better tertiary outcomes (depression [59, 60]), self-rated health [61], internal locus of control [62], and health-related quality of life [63, 64]) which is clinically important in this clinical population. For example, in a prior study of 46 PWH randomized to SOP training or a no-contact control group, improvements were observed on the Timed IADL (Instrumental Activities of Daily Living) test after SOP training [32].

As SOP training may improve locus of control, it may also provide reassurance to PWH who are concerned about their cognition as they age. In a sample of 85 PWH who were told of their HAND diagnosis, a qualitative assessment of their reactions included a variety of themes including *Sadness, Anxiety*, and *Concern* [65]. Interestingly, other themes emerged as well including *Confirmation* (that validated they were experiencing cognitive issues), *Knowledge Seeking*, and *Desire to Improve*. Given that many PWH may want to try something safe and effective to protect their cognition, SOP training may be such an approach. In a recent study of individualized-cognitive training in 109 PWH with HAND, those who went through cognitive training reported feeling their cognition improved, which may unto itself reduce stress and improve locus of control [66].

Unfortunately, in the Think Fast Study as we followed up with analyses examining the impact of SOP training on everyday functioning (Aim 2; [34]) and on quality of life indicators (Aim 3 [35]) in separate publications, in our sample we found SOP training did not improve locus of control or reduce depressive symptomatology. It is not clear why such findings were not observed. In prior studies in which locus of control was improved in community-dwelling older adults, they may have been less resistant to improvement; meanwhile, PWH may have other factors that may interfere with improvement in depression and locus of control such as also coping with HIV-related stigma and factors related to social determinants of health (i.e., lower education and income) [67]. Yet, in our aim investigating the effect of SOP training on everyday functioning, we did observe some improvement in measures of medication adherence [34]; this is an important finding given the importance of medication adherence has on survival and improved health outcomes in PWH.

### Strengths and Limitations

Several study strengths are noted. First, we employed commercially available, standardized cognitive training modules which confirms that participants received an identical training, ensuring replication [36]. Second, we used standardized normed cognitive measures and accepted protocols for classifying HAND [36]. Third, we followed participants for up to 2 years, filling a major gap in the HIV cognitive intervention literature [9]. Fourth, we had a large and diverse sample in terms of race and comorbidities; thus, the findings are more ecologically valid and representative of the larger HIV population [36]. And fifth, regarding substance use, this study is ecologically valid. The rate of substance use in PWH is generally higher than in the general population. In a large cohort of 10,652 PWH linked to care at seven sites, Hartzler et al. [68] found the prevalence of substance use disorder was at 48% in the United States. Thus, our sample at 44% seems consistent with the literature. As seen in Table 1, in greater transparency we did document substance use by: (1) alcohol frequency, (2) number of drinks on a drinking day, (3) currently using tobacco, and (4) cigarettes per day. As can be seen, the rates were very consistent across the three groups. As for other types of substances we tested for in the UTOX + (marijuana, amphetamine, methamphetamine, cocaine, and opioids), most of this was marijuana. As our study did not provide any type of substance use treatment or education, we do not suspect that the rate of substance use would have changed significantly over time. In a follow-up article, it may be of value to examine whether substance use influences the effect of SOP training on UFOV® performance and other cognitive outcomes.

Likewise, four study limitations are noted. First, methodologically pure SOP training does not exist. The exercises are designed to maximize improvement on SOP, but there are other cognitive components that are likely being utilized to a lesser extent to complete the exercises. Second, we consider the attrition rate for this study—47.22% over 2 years—to be relatively high compared to attrition rates (e.g., 19.3%, 21.4% 25%) in cognitive training studies with HIV over short (e.g., 5–6 weeks) amounts of time [9]. But since this is a 2-year study, there are more opportunities over time for participants to drop out; as can be seen at posttest (Fig. 1), the attrition rate was 19.44% reflective of these studies. Yet, the attrition could have resulted from several factors, and an important one being transportation. In this same sample reported in a different article, 37.5% of participants indicated that transportation was an issue for them in adhering to daily appointments [47]. Third, this study did include other cognitive variables from several other domains (e.g., executive functioning, learning/memory) that were not examined in this article. For context, although we found SOP training improved UFOV® performance, SOP training did not transfer to improvement in other cognitive domains in the Think Fast Study [33]. This finding suggests that the type of SOP training we used in this case was more aligned to improve a very specific cognitive ability (i.e., useful field of view) rather than generalized improvement to other types of cognitive functioning (i.e., verbal memory). And fourth, the key to randomization was held by the study principal investigator and statistician, and not by a third party at arms-length from the study employees and participants.

In conclusion, this is the first 2-year longitudinal study examining cognitive training in a large sample of middle-aged and older PWH cognitively at risk. The effects of SOP training are encouraging. As discussed, in examining SOP training in other study aims investigating everyday functioning and quality of life outcomes, we found some support for SOP training improving medication adherence but it was not effective in reducing depressive symptomatology or improving locus of control [34, 35]. Given the impact SOP training has on other relevant outcomes (i.e., driving and driving simulator outcomes) in other populations, an important future research step will be to examine the transfer effects to other clinically relevant outcomes among older PWH.

## Supplementary Information

Below is the link to the electronic supplementary material.

Supplementary file1 (DOCX 40 KB)

## Data Availability

Data are available upon request. Data are stored on a server at the School of Nursing, University of Alabama at Birmingham, Birmingham, Alabama. Geographic Information: Data were collected in Birmingham, Alabama.

## Declarations

### Research Involving Human and Animal Participants

Written informed consent was obtained using procedures approved by the investigational review board at each of the collaborating institutions. All procedures performed in this study involving human participants were in accordance with the ethical standards of the institutional and/or national research committee and with the 1964 Helsinki declaration and its later amendments or comparable ethical standards.

## References

[1] Goodkin K, Miller E, Cox C, et al. Effect of ageing on neurocognitive function by stage of HIV infection: evidence from the multicenter AIDS Cohort Study. Lancet HIV. 2017;4(9):e411–22. 10.1016/S2352-3018(17)30098-X. 28716545 10.1016/S2352-3018(17)30098-X PMC5753579

[2] Vance DE, Fazeli PL, Ball DA, et al. Cognitive functioning and driving simulator performance in middle-aged and older adults with HIV. J Assoc Nurses AIDS Care. 2014;25(2):e11-26. 10.1016/j.jana.2013.12.001. 24513104 10.1016/j.jana.2013.12.001 PMC3939674

[3] Vance DE, Fazeli PL, Gakumo CA. The impact of neuropsychological performance on everyday functioning between older and younger adults with and without HIV. J Assoc Nurses AIDS Care. 2013;24(2):112–25. 10.1016/j.jana.2012.05.002. 22943982 10.1016/j.jana.2012.05.002 PMC3515709

[4] Lin F, Chin D-G, Vance DE, et al. Longitudinal relationships between subjective fatigue, cognitive function, and everyday functioning in old age. Int Psychogeriatr. 2013;25(2):275–85. 10.1017/S1041610212001718. 23083533 10.1017/S1041610212001718 PMC3552486

[5] Center for Disease Control and Prevention. HIV Surveilance Report, 2018 (updated). 2020. http://www.cdc.gov/hiv/library/reports/hiv-surveillance.html.

[6] Smit M, Brinkman K, Geerlings S, et al. Future challenges for clinical care of an ageing population infected with HIV: a modelling study. Lancet Infect Dis. 2015;15(7):810–8. 10.1016/S1473-3099(15)00056-0. 26070969 10.1016/S1473-3099(15)00056-0 PMC4528076

[7] Vance DE. Aging with HIV: clinical considerations for an emerging population. Am J Nurs. 2010;110(3):42–7. 10.1097/01.NAJ.0000368952.80634.4. 20179457 10.1097/01.NAJ.0000368952.80634.42 PMC4571195

[8] Lampit A, Hallock H, Valenzuela M. Computerized cognitive training in cognitively healthy older adults: a systematic review and meta-analysis of effect modifiers. PLoS Med. 2014;11(11): e1001756. 10.1371/journal.pmed.1001756. 25405755 10.1371/journal.pmed.1001756 PMC4236015

[9] Vance DE, Fazeli PL, Cheatwood J, et al. Computerized cognitive training for the neurocognitive complications of HIV infection: a systematic review. J Assoc Nurses AIDS Care. 2019;30(1):51–72. 10.1097/JNC.0000000000000030. 30586083 10.1097/JNC.0000000000000030

[10] Vance DE, McNees P, Meneses K. Technology, cognitive remediation, and nursing: directions for successful cognitive aging. J Gerontol Nurs. 2009;35(2):50–6. 10.3928/00989134-20090201-09. 19263921 10.3928/00989134-20090201-09

[11] Vance DE, Fazeli PL, Azuero A, et al. Can individualized-targeted computerized cognitive training benefit adults with HIV-associated neurocognitive disorder? The Training on Purpose Study (TOPS). AIDS Behav. 2021;25(12):3898–908. 10.1007/s10461-021-03230-y. 33733311 10.1007/s10461-021-03230-y PMC11951421

[12] Ball K, Berch DB, Helmers KF, et al. Effects of cognitive training interventions with older adults: a randomized controlled trial. JAMA. 2002;288(18):2271–81. 12425704 10.1001/jama.288.18.2271 PMC2916176

[13] Rebok GW, et al. Ten-year effects of the advanced cognitive training for independent and vital elderly cognitive training trial on cognition and everyday functioning in older adults. J Am Geriatr Soc. 2014;62(1):16–24. 10.1001/jama.288.18.2271. 24417410 10.1111/jgs.12607 PMC4055506

[14] Edwards JD, Xu H, Clark DO, et al. Speed of processing training results in lower risk of dementia. Alzheimers Dement (N Y). 2017;3(4):603–11. 10.1016/j.trci.2017.09.002. 29201994 10.1016/j.trci.2017.09.002 PMC5700828

[15] Dastgheyb RM, Buchholz AS, Fitzgerald KC, et al. Patterns and predictors of cognitive function among virally suppressed women with HIV. Front Neurol. 2021;12: 604984. 10.3389/fneur.2021.604984. 33679577 10.3389/fneur.2021.604984 PMC7928382

[16] Fitzgerald KC, Maki PM, Xu Y, et al. Factors predicting detrimental change in declarative memory among women with HIV: a study of heterogeneity in cognition. Front Psychol. 2020;11: 548521. 10.3389/fpsyg.2020.548521. 33178064 10.3389/fpsyg.2020.548521 PMC7594511

[17] Reger M, Welsch R, Razani J, et al. A meta-analysis of the neuropsychological sequelae of HIV infection. J Int Neuropsychol Soc. 2002;8(3):410–24. 10.1017/s1355617702813212. 11939699 10.1017/s1355617702813212

[18] Waldrop D, Irwin C, Nicholson WC, et al. The intersection of cognitive ability and HIV: a review of the state of the nursing science. J Assoc Nurses AIDS Care. 2021;32(3):306–21. 10.1097/JNC.0000000000000232. 33449578 10.1097/JNC.0000000000000232 PMC8091162

[19] Fazeli PL, Marceaux JC, Vance DE, et al. Predictors of cognition in adults with HIV: implications for nursing practice and research. J Neurosci Nurs. 2011;43(1):36–50. 10.1097/jnn.0b013e3182029790. 21338043 10.1097/jnn.0b013e3182029790 PMC3039887

[20] Marcotte TD, Deutsch R, McCutchan JA, et al. Prediction of incident neurocognitive impairment by plasma HIV RNA and CD4 levels early after HIV seroconversion. Arch Neurol. 2003;60(10):1406–12. 10.1001/archneur.60.10.1406. 14568811 10.1001/archneur.60.10.1406

[21] Mindt MR, Miranda C, Arentoft A, et al. Aging and HIV/AIDS: neurocognitive implications for older HIV-positive Latina/o adults. Behav Med. 2014;40(3):116–23. 10.1080/08964289.2014.914464. 25090364 10.1080/08964289.2014.914464 PMC5584638

[22] Moore DJ, Letendre SL, Morris S, et al. Neurocognitive functioning in acute or early HIV infection. J Neurovirol. 2011;17(1):50–7. 10.1007/s13365-010-0009-y. 21165782 10.1007/s13365-010-0009-y PMC3032208

[23] Moore DJ, Masliah E, Rippeth JD, et al. Cortical and subcortical neurodegeneration is associated with HIV neurocognitive impairment. AIDS. 2006;20(6):879–87. 10.1097/01.aids.0000218552.69834.00. 16549972 10.1097/01.aids.0000218552.69834.00

[24] Salthouse TA. The processing-speed theory of adult age differences in cognition. Psychol Rev. 1996;103(3):403–28. 10.1037/0033-295x.103.3.403. 8759042 10.1037/0033-295x.103.3.403

[25] Birren JE. Age changes in speed of behavior: its central nature and physiological correlates. In: Welford AT, Birren JE, editor. Behavior, aging and the nervous system: Biological determinants of speed and behavior. Springfield; 1965.

[26] Fristoe NM, Salthouse TA, Woodard JL. Examination of age-related deficits on the Wisconsin Card Sorting Test. Neuropsychology. 1997;11(3):428–36. 10.1037/0894-4105.11.3.428. 9223147 10.1037//0894-4105.11.3.428

[27] Cerella J. Generalized slowing in Brinley plots. J Gerontol. 1994;49(2):P65-71. 10.1093/geronj/49.2.p65. 8126361 10.1093/geronj/49.2.p65

[28] Cerella J, Hale S. The rise and fall in information-processing rates over the life span. Acta Psychol (Amst). 1994;86(2–3):109–97. 10.1016/0001-6918(94)90002-7. 7976466 10.1016/0001-6918(94)90002-7

[29] Vance DE. Speed of processing in older adults: a cognitive overview for nursing. J Neurosci Nurs. 2009;41(6):290–7. 10.1097/jnn.0b013e3181b6bed. 19998680 10.1097/jnn.0b013e3181b6beda

[30] Fellows RP, Byrd DA, Morgello S. Effects of information processing speed on learning, memory, and executive functioning in people living with HIV/AIDS. J Clin Exp Neuropsychol. 2014;36(8):806–17. 10.1080/13803395.2014.943696. 25111120 10.1080/13803395.2014.943696 PMC4338860

[31] Doyle KL, Morgan EE, Morris S, et al. Real-world impact of neurocognitive deficits in acute and early HIV infection. J Neurovirol. 2013;19(6):565–73. 10.1007/s13365-013-0218-2. 24277439 10.1007/s13365-013-0218-2 PMC3865175

[32] Vance DE, Fazeli PL, Ross LA, et al. Speed of processing training with middle-age and older adults with HIV: a pilot study. J Assoc Nurses AIDS Care. 2012;23(6):500–10. 10.1016/j.jana.2012.01.005. 22579081 10.1016/j.jana.2012.01.005 PMC3422374

[33] Vance DE, Fazeli PL, Azuero A, et al. A 2-year longitudinal randomized controlled trial examining the transfer of speed of processing training to secondary cognitive domains in middle-aged and older adults with HIV-associated neurocognitive disorder: results of the Think Fast Study. Clin Neuropsychol. 2023;38(2):471–92. 10.1080/13854046.2023.2212867. 37191339 10.1080/13854046.2023.2212867 PMC10651797

[34] Vance DE, Fazeli PL, Azuero A, et al. Two-year clinical trial examining the effects of speed of processing training on everyday functioning in adults with human immunodeficiency virus-associated neurocognitive disorder (HAND) and borderline HAND in the U.S. Deep South: findings of the Think Fast Study. Appl Neuropsychol Adult. 2023. 10.1080/23279095.2023.2209900. 37200482 10.1080/23279095.2023.2209900 PMC10656361

[35] Vance DE, Fazeli PL, Andres A, et al. A 2-year randomized clinical trial examining the effects of speed of processing cognitive training on quality of life indicators in adults with HIV-associated neurocognitive disorder in Birmingham, Alabama: results of the Think Fast Study. J Assoc Nurses AIDS Care. 2024;35(2):104–21. 10.1097/JNC.0000000000000449. 38949906 10.1097/JNC.0000000000000449 PMC11217600

[36] Vance DE, Fazeli PL, Shacka J, et al. Testing a computerized cognitive training protocol in adults aging with HIV-associated neurocognitive disorders: randomized controlled trial rationale and protocol. JMIR Res Protoc. 2017;6(4): e68. 10.2196/resprot.6625. 28446421 10.2196/resprot.6625 PMC5422019

[37] Radloff LS. The CES-D Scale: a self-report depression scale for research in the general population. Appl Psychol Meas. 1977;1(3):385–440.

[38] Blackstone K, Moore DJ, Franklin DR, et al. Defining neurocognitive impairment in HIV: deficit scores versus clinical ratings. Clin Neuropsychol. 2012;26(6):894–908. 10.1080/13854046.2012.694479. 22708483 10.1080/13854046.2012.694479 PMC3848322

[39] Heaton RK, Clifford DB, Franklin DR Jr, et al. HIV-associated neurocognitive disorders persist in the era of potent antiretroviral therapy: CHARTER Study. Neurology. 2010;75(23):2087–96. 10.1212/WNL.0b013e318200d727. 21135382 10.1212/WNL.0b013e318200d727 PMC2995535

[40] Woods SP, Rippeth JD, Frol ABF, et al. Interrater reliability of clinical ratings and neurocognitive diagnoses in HIV. J Clin Exp Neuropsychol. 2004;26(6):759–78. 10.1080/13803390490509565. 15370374 10.1080/13803390490509565

[41] Grant I, Franklin DR Jr, Deutsch R, et al. Asymptomatic HIV-associated neurocognitive impairment increases risk for symptomatic decline. Neurology. 2014;82(23):2055–62. 10.1212/WNL.0000000000000492. 24814848 10.1212/WNL.0000000000000492 PMC4118496

[42] Ball K, Owsley C, Sloane ME, et al. Visual attention problems as a predictor of vehicle crashes in older drivers. Optom Vis Sci. 1993;34(11):3110–23. 8407219

[43] Wood JM, Owsley C. Useful field of view test. Gerontology. 2014;60(4):315–8. 10.1159/000356753. 24642933 10.1159/000356753 PMC4410269

[44] Edwards JD, Vance DE, Wadley VG, et al. Reliability and validity of useful field of view test scores as administered by personal computer. J Clin Exp Neuropsychol. 2005;27(5):529–43. 10.1080/13803390490515432. 16019630 10.1080/13803390490515432

[45] Wood JM, Chaparro A, Lacherez P, et al. Useful field of view predicts driving in the presence of distracters. Optom Vis Sci. 2012;89(4):373–81. 10.1097/OPX.0b013e31824c17ee. 22366710 10.1097/OPX.0b013e31824c17ee

[46] Vance DE, Wright MA. Positive and negative neuroplasticity: implications for age-related cognitive declines. J Gerontol Nurs. 2009;35(6):11–7. 10.3928/00989134-20090428-02. 19537290 10.3928/00989134-20090428-02

[47] Pope CN, Stavrinos D, Fazeli PL, et al. Transportation barriers and health-related quality of life in a sample of middle-aged and older adults living with HIV in the deep South. AIDS Behav. 2022;26(7):2148–58. 10.1007/s10461-021-03560-x. 35066731 10.1007/s10461-021-03560-x PMC8783768

[48] Cohen J. Statistical power analysis for the behaviroal sciences. 2nd ed. Milton Park: Routledge; 1988. 10.4324/9780203771587.

[49] Groenwold RH, Donders AR, Roes KC, et al. Dealing with missing outcome data in randomized trials and observational studies. Am J Epidemiol. 2012;175(3):210–7. 10.1093/aje/kwr302. 22262640 10.1093/aje/kwr302

[50] Benjamini Y, Hochberg Y. Controlling the false discovery rate: a practical and powerful approach to multiple testing. J R Stat Soc Ser B (Methodoloigcal). 1995;57(1):289–300. 10.1111/j.2517-6161.1995.tb02031.x.

[51] Team, R.C. A language and environment for statistical computing. 2021, Vienna, Austria: R Foundation for Statistical Computing

[52] Vance DE, Dawson J, Wadley VG, et al. The accelerate study: the longitudinal effect of speed of processing training on cognitive performance of older adults. Rehabil Psychol. 2007;51(1):89–96. 10.1037/0090-5550.52.1.89.

[53] O’Brien JL, Edward JD, Maxfield ND, et al. Cognitive training and selective attention in the aging brain: an electrophysiological study. Clin Neurophysiol. 2013;124(11):2198–208. 10.1016/j.clinph.2013.05.012. 23770088 10.1016/j.clinph.2013.05.012

[54] Vance DE, Mugavero M, Willig J, et al. Aging with HIV: a cross-sectional study of comorbidity prevalence and clinical characteristics across decades of life. J Assoc Nurses AIDS Care. 2011;22(1):17–25. 10.1016/j.jana.2010.04.002. 20471864 10.1016/j.jana.2010.04.002

[55] Effros RB, Fletcher CV, Gebo K, et al. Aging and infectious diseases: workshop on HIV infection and aging: what is known and future research directions. Clin Infect Dis. 2008;47(4):542–53. 10.1086/590150. 18627268 10.1086/590150 PMC3130308

[56] Gebo KA, Justice A. HIV infection in the elderly. Curr Infect Dis Rep. 2009;11(3):246–54. 10.1007/s11908-009-0036-0. 19366568 10.1007/s11908-009-0036-0 PMC3129647

[57] Vance DE, Fazeli PL, Moneyham L, et al. Assessing and treating forgetfulness and cognitive problems in adults with HIV. J Assoc Nurses AIDS Care. 2013;24(1 Suppl):S40-60. 10.1016/j.jana.2012.03.006. 23290376 10.1016/j.jana.2012.03.006 PMC3591816

[58] Vance DE, Wadley VG, Crowe MG, et al. Cognitive and everyday functioning in older and younger adults with and without HIV. Clin Gerontol. 2011;34(5):413–26. 10.1080/07317115.2011.588545. 22563140 10.1080/07317115.2011.588545 PMC3342698

[59] Wolinsky FD, Mahncke HW, Vander Weg MW, et al. The ACTIVE cognitive training interventions and the onset of and recovery from suspected clinical depression. J Gerontol B Psychol Sci Soc Sci. 2009;64(5):577–85. 10.1093/geronb/gbp061. 19617456 10.1093/geronb/gbp061 PMC2728092

[60] Wolinsky FD, Vander Weg MW, Martin R, et al. The effect of speed-of-processing training on depressive symptoms in ACTIVE. J Gerontol A Biol Sci Med Sci. 2009;64(4):468–72. 10.1093/gerona/gln044. 19181719 10.1093/gerona/gln044 PMC2657170

[61] Wolinsky FD, Mahncke HW, Vander Weg MW, et al. Speed of processing training protects self-rated health in older adults: enduring effects observed in the multi-site ACTIVE randomized controlled trial. Int Psychogeriatr. 2010;22(3):470–8. 10.1017/S1041610209991281. 20003628 10.1017/S1041610209991281 PMC2848284

[62] Wolinsky FD, Vander Weg MW, Martin R, et al. Does cognitive training improve internal locus of control among older adults? J Gerontol B Psychol Sci Soc Sci. 2010;65(5):591–8. 10.1093/geronb/gbp117. 20008028 10.1093/geronb/gbp117 PMC2920943

[63] Wolinsky FD, Unverzagt FW, Smith DM, et al. The effects of the ACTIVE cognitive training trial on clinically relevant declines in health-related quality of life. J Gerontol B Psychol Sci Soc Sci. 2006;61(5):S281–7. 10.1093/geronb/61.5.s281. 16960242 10.1093/geronb/61.5.s281

[64] Wolinsky FD, Unverzagt FW, Smith DM, et al. The ACTIVE cognitive training trial and health-related quality of life: protection that lasts for 5 years. J Gerontol A Biol Sci Med Sci. 2006;61(12):1324–9. 10.1093/gerona/61.12.1324. 17234829 10.1093/gerona/61.12.1324

[65] Vance DE, Jensen M, Tende F, et al. Informing adults with HIV of cognitive performance deficits indicative of HIV-associated neurocognitive disorder: a content analysis. J Psychosoc Nurs Ment Health Serv. 2019;57(12):48–55. 10.3928/02793695-20190821-03. 31437282 10.3928/02793695-20190821-03 PMC13277363

[66] Byun JY, Azuero A, Fazeli PL, et al. Perceived improvement and satisfaction with training after individualized-targeted computerized cognitive training in adults with HIV-associated neurocognitive disorder living in Alabama: a descriptive cross-sectional study. J Assoc Nurses AIDS Care. 2022;33(5):581–6. 10.1097/JNC.0000000000000333. 35363623 10.1097/JNC.0000000000000333 PMC13309983

[67] Slater LZ, Moneyham L, Vance DE, et al. The multiple stigma experience and quality of life in older gay men with HIV. J Assoc Nurses AIDS Care. 2015;26(1):24–35. 10.1016/j.jana.2014.06.007. 25249266 10.1016/j.jana.2014.06.007

[68] Hartzler B, Dombrowski JC, Crane HM, et al. Prevalence and predictors of substance use disorders among HIV care enrollees in the United States. AIDS Behav. 2017;21(4):1138–48. 10.1007/s10461-016-1584-6. 27738780 10.1007/s10461-016-1584-6 PMC6089366

