# Supplementary material for: A 2-Year Longitudinal Randomized Control Trial of Speed of Processing Cognitive Training in Aging Adults with HIV-Associated Neurocognitive Disorder: Results of the Think Fast Study
Source: AIDS Behav. 2024 Jul 1;28(10):3300–14. doi: 10.1007/s10461-024-04409-9 (PMC11427548; doi:10.1007/s10461-024-04409-9)
Supplement: Supplementary file 1 — Supplementary file1 (DOCX 40 KB) [file 10461_2024_4409_MOESM1_ESM.docx]

**Supplemental Table 1**

Study Attrition Comparisons of Baseline Participant Characteristics of Those Who Dropped Out and Those Who Remained in the Study with Data for All Follow-Up Time-Points in a Randomized Control Trial of Speed of Processing Training in Adults with HIV

| Variable | Dropped Out (*n*=102) | | Remained in Study (*n*=114) | | *Effect size* |
| --- | --- | --- | --- | --- | --- |
|  | *n* (%) | Mean (*SD*) | *n* (%) | Mean (*SD*) |  |
| Age |  | 50.42 (6.71) |  | 51.54 (6.34) | \|d\|=0.17 |
| Gender |  |  |  |  | V=0.01 |
| Female | 39 (38.24%) |  | 43 (37.72%) |  |  |
| Male | 63 (61.76%) |  | 71 (62.28%) |  |  |
| Race/Ethnicity |  |  |  |  | V=0.06 |
| Non-white | 82 (80.39%) |  | 97 (85.09%) |  |  |
| White | 20 (19.61%) |  | 17 (14.91%) |  |  |
| Education (years) |  | 12.16 (2.44) |  | 12.62 (2.09) | \|d\|=0.21 |
| Household income ($10K) |  | 1.89 (1.70) |  | 1.78 (1.27) | \|d\|=0.07 |
| Years diagnosed with HIV |  | 15.58 (8.49) |  | 16.47 (8.53) | \|d\|=0.1 |
| Current CD4+ T lymphocyte count/mm^3^ |  | 634.74 (369.63) |  | 664.30 (383.54) | \|d\|=0.08 |
| Nadir CD4+ T lymphocyte count/mm^3^ |  | 311.46 (303.62) |  | 237.01 (247.74) | \|d\|=0.27 |
| No. of prescribed medications |  | 6.15 (4.53) |  | 7.32 (4.70) | \|d\|=0.25 |
| Prescribed ART |  |  |  |  | V=0.17 |
| No | 8 (7.84%) |  | 1 (0.88%) |  |  |
| Yes | 87 (85.29%) |  | 105 (92.11%) |  |  |
| Unknown | 7 (6.86%) |  | 8 (7.02%) |  |  |
| CES-Depression |  | 17.63 (10.67) |  | 19.06 (11.45) | \|d\|=0.13 |
| Locus of Control |  | 28.18 (6.35) |  | 27.25 (5.94) | \|d\|=0.15 |
| Alcohol use frequency |  |  |  |  | V=0.12 |
| Never | 49 (48.51%) |  | 43 (37.72%) |  |  |
| Monthly or less | 21 (20.79%) |  | 26 (22.81%) |  |  |
| Two to four times a month | 13 (12.87%) |  | 22 (19.30%) |  |  |
| Twice weekly or more | 18 (17.82%) |  | 23 (20.18%) |  |  |
| Currently using tobacco | 58 (56.86%) |  | 61 (53.51%) |  | V=0.03 |
| UTOX+ | 44 (44.44%) |  | 49 (44.14%) |  | V=0 |
| Baseline Outcomes: |  |  |  |  |  |
| Global Clinical Rating Scale |  | 5.16 (1.09) |  | 5.27 (1.10) | \|d\|=0.11 |
| HAND |  |  |  |  | V=0.1 |
| Impaired | 66 (64.71%) |  | 84 (73.68%) |  |  |
| Normal | 36 (35.29%) |  | 30 (26.32%) |  |  |
| UFOVTOTAL (ms) |  | 748.14 (316.08) |  | 763.26 (326.15) | \|d\|=0.05 |
| UFOV1 (ms) |  | 38.98 (58.50) |  | 42.54 (59.45) | \|d\|=0.06 |
| UFOV2 (ms) |  | 113.49 (109.30) |  | 112.04 (107.22) | \|d\|=0.01 |
| UFOV3 (ms) |  | 209.84 (111.36) |  | 221.67 (125.13) | \|d\|=0.1 |
| UFOV4 (ms) |  | 385.82 (111.54) |  | 387.02 (104.32) | \|d\|=0.01 |
| RISCAT |  | 1.76 (0.9 = 9) |  | 1.87 (1.08) | \|d\|=0.1 |
| Study group |  |  |  |  | V=0.11 |
| Control | 40 (39.22%) |  | 33 (28.95%) |  |  |
| 10 Hours training | 30 (29.41%) |  | 40 (35.09%) |  |  |
| 20 Hours training | 32 (31.37%) |  | 41 (35.96%) |  |  |
| Notes. \|d\| = absolute value of Cohen’s d; V=Cramer’s V; ART=antiretroviral therapy; CES-Depression=Center for  Epidemiological Studies Depression Scale; ms=milliseconds in which correctly responded; no.=number; RISCAT=  UFOV risk category; SD=standard deviation; UFOV=Useful Field of View; UTOX+=urine toxicology screen indicating  a positive result; $10K=ten thousand dollars. Cohen’s d: ~0.2 small, ~0.5 medium, ~0.8 large. In this table, Cramer’s V:  ~0.1 small, ~0.3 medium, ~0.5 large. | | | | | |

**Supplemental Table 2**

Subgroup Analysis: Baseline Demographic and Clinical Characteristics of 108 Participants with HIV with Greater UFOV^®^ Risk (RISCAT > 2 at Baseline) in a Randomized Controlled Trial of Speed of Processing Training Dosages Compared to a Contact Control Group

| Variable | Contact Control Group (*n*=37) | | 10-hour Cognitive Training (*n*=36) | | 20-hour Cognitive Training (*n*=35) | | *Effect size* |
| --- | --- | --- | --- | --- | --- | --- | --- |
|  | *n* (%) | Mean (*SD*) | *n* (%) | Mean (*SD*) | *n* (%) | Mean (*SD*) |  |
| Age |  | 51.08 (6.46) |  | 51.97 (6.43) |  | 53.66 (5.87) | R2=0.03 |
| Gender |  |  |  |  |  |  | V=0.15 |
| Female | 19 (51.35%) |  | 12 (33.33%) |  | 15 (42.86%) |  |  |
| Male | 18 (48.65%) |  | 24 (66.67%) |  | 20 (57.14%) |  |  |
| Race/Ethnicity |  |  |  |  |  |  | V=0.05 |
| Non-white | 34 (91.89%) |  | 34 (94.44%) |  | 32 (91.43%) |  |  |
| White | 3 (8.11%) |  | 2 (5.56%) |  | 3 (8.57%) |  |  |
| Education (years) |  | 11.59 (2.02) |  | 12.08 (1.87) |  | 12.29 (1.60) | R2=0.03 |
| Household income ($10K) |  | 1.43 (0.60) |  | 1.58 (1.18) |  | 1.69 (1.16) | R2=0.01 |
| Years diagnosed with HIV |  | 15.67 (8.83) |  | 14.29 (8.38) |  | 19.42 (7.14) | R2=0.07 |
| Current CD4+ T lymphocyte count/mm^3^ |  | 719.12 (368.78) |  | 624.48 (352.55) |  | 589.43 (370.12) | R2=0.02 |
| Nadir CD4+ T lymphocyte count/mm^3^ |  | 312.62 (285.77) |  | 237.45 (224.02) |  | 237.23 (292.78) | R2=0.02 |
| No.of prescribed medications |  | 7.05 (4.39) |  | 6.28 (3.57) |  | 7.83 (6.02) | R2=0.02 |
| Prescribed ART |  |  |  |  |  |  | V=0.14 |
| No | 3 (8.11%) |  | 1 (2.78%) |  | 1 (2.86%) |  |  |
| Yes | 31 (83.78%) |  | 30 (83.33%) |  | 33 (94.29%) |  |  |
| Unknown | 3 (8.11%) |  | 5 (13.89%) |  | 1 (2.86%) |  |  |
| CES-Depression |  | 20.27 (10.88) |  | 20.03 (10.82) |  | 16.29 (9.67) | R2=0.03 |
| Locus of Control |  | 25.22 (6.70) |  | 26.14 (6.34) |  | 26.83 (6.30) | R2=0.01 |
| Alcohol use frequency |  |  |  |  |  |  | V=0.08 |
| Never | 15 (40.54%) |  | 17 (47.22%) |  | 17 (50.00%) |  |  |
| Monthly or less | 9 (24.32%) |  | 8 (22.22%) |  | 8 (23.53%) |  |  |
| Two to four times a month | 6 (16.22%) |  | 4 (11.11%) |  | 5 (14.71%) |  |  |
| Twice weekly or more | 7 (18.92%) |  | 7 (19.44%) |  | 4 (11.76%) |  |  |
| No. of drinks on a drinking day |  | 3.27 (2.20) |  | 3.13 (2.40) |  | 2.71 (2.23) | R2=0.01 |
| Currently using tobacco | 22 (59.46%) |  | 23 (63.89%) |  | 16 (45.71%) |  | V=0.15 |
| Cigarettes per day |  | 9.77 (6.57) |  | 8.36 (5.88) |  | 10.29 (7.69) | R2=0.02 |
| UTOX+ | 14 (37.84%) |  | 13 (36.11%) |  | 17 (51.52%) |  | V=0.1 |
| Study Activities: |  |  |  |  |  |  |  |
| Hours logged in training activities |  | 8.38 (3.62) |  | 8.86 (2.96) |  | 18.59 (4.79) | R2=0.6 |
| % of prescribed training activity hours  logged |  | 83.86 (36.01) |  | 88.49 (29.27) |  | 93.07 (23.88) | R2=0.02 |
| Hours of computerized cognitive  training |  | NA (NA) |  | 7.37 (2.93) |  | 13.95 (5.10) | R2=0.39 |
| Cognitive Function: |  |  |  |  |  |  |  |
| Global Function T |  | 43.36 (5.22) |  | 42.74 (5.47) |  | 43.33 (4.49) | R2=0 |
| Global Clinical Rating Scale |  | 5.33 (1.16) |  | 5.17 (1.15) |  | 5.32 (1.01) | R2=0 |
| HAND |  |  |  |  |  |  | V=0.02 |
| Impaired | 24 (72.73%) |  | 30 (75.00%) |  | 30 (73.17%) |  |  |
| Normal | 9 (27.27%) |  | 10 (25.00%) |  | 11 (26.83%) |  |  |
| Baseline Outcomes: |  |  |  |  |  |  |  |
| UFOVTOTAL (ms) |  | 943.38 (287.97) |  | 940.47 (322.93) |  | 1007.26 (314.56) | R2=0.01 |
| UFOV1 (ms) |  | 51.54 (63.02) |  | 66.17 (86.33) |  | 73.54 (80.47) | R2=0.01 |
| UFOV2 (ms) |  | 179.49 (96.93) |  | 180.22 (110.64) |  | 205.34 (112.38) | R2=0.01 |
| UFOV3 (ms) |  | 282.84 (126.90) |  | 266.03 (127.86) |  | 289.20 (118.18) | R2=0.01 |
| UFOV4 (ms) |  | 429.51 (102.00) |  | 428.06 (78.56) |  | 439.17 (88.97) | R2=0 |
| RISCAT |  | 2.49 (0.73) |  | 2.58 (0.94) |  | 2.86 (0.97) | R2=0.03 |
| Follow-up data collection |  |  |  |  |  |  | V=0.03 |
| Post | 29 (78.38%) |  | 30 (83.33%) |  | 31 (88.57%) |  |  |
| Year 1 | 23 (62.16%) |  | 29 (80.56%) |  | 25 (71.43%) |  |  |
| Year 2 | 18 (48.65%) |  | 21 (58.33%) |  | 19 (54.29%) |  |  |

*Notes.* R2=R-squared; V=Cramer's V; ART=antiretroviral therapy; CES-Depression=Center for Epidemiological Studies Depression Scale; ms=milliseconds in which correctly responded; no.=number; RISCAT=UFOV risk category; SD=standard deviation; UFOV=Useful Field of View; UTOX+=urine toxicology screen indicating a positive result; SD=standard deviation; $10K=ten thousand dollars. R2: ~0.02 small, ~0.13 medium, ~0.26 large. In this table, Cramer's V: ~0.07 small, ~0.21 medium, ~0.35 large.

**Supplemental Table 3**

Subgroup Analysis: A Randomized Controlled Trial of 108 Participants with HIV with Greater UFOV^®^ Risk (RISCAT > 2 at Baseline) Assigned to Speed of Processing Training Dosages or a Contact Control Group Comparing UFOV^®^ Treatment Outcomes by Group at Posttest, Year 1, and Year 2 Follow-up

| **Outcomes by group and between-group contrasts** | **POST** | | | **Year 1** | | | **YEAR 2** | | |
| --- | --- | --- | --- | --- | --- | --- | --- | --- | --- |
|  | Mean (SE) | P | d (95% CI) | Mean (SE) | P | d (95% CI) | Mean (SE) | P | d (95% CI) |
| UFOVTOTAL (ms) |  |  |  |  |  |  |  |  |  |
| Control | 873.45 (30.92) | - | - | 839.7 (41.37) | - | - | 779.74 (67.91) | - | - |
| 10 Hrs. | 744.79 (29.83) | - | - | 797.98 (36.93) | - | - | 749.25 (59.62) | - | - |
| 20 Hrs. | 643.22 (29.47) | - | - | 712.55 (39.83) | - | - | 660.38 (62.95) | - | - |
| Control vs. 10 Hrs. | 128.66 (43.02) | 0.003 | 0.42 (0.14, 0.7) | 41.71 (55.46) | 0.453 | 0.14 (-0.22, 0.49) | 30.49 (90.38) | 0.737 | 0.1 (-0.48, 0.68) |
| Control vs. 20 Hrs. | 230.23 (42.87) | <0.001 | 0.75 (0.45, 1.04) | 127.14 (57.51) | 0.028 | 0.41 (0.04, 0.78) | 119.37 (92.65) | 0.201 | 0.39 (-0.21, 0.99) |
| 10 Hrs. vs. 20 Hrs. | 101.57 (41.97) | 0.016 | 0.33 (0.06, 0.6) | 85.43 (54.44) | 0.118 | 0.28 (-0.07, 0.63) | 88.87 (86.77) | 0.309 | 0.29 (-0.27, 0.85) |
| UFOV1 (ms) |  |  |  |  |  |  |  |  |  |
| Control | 41.44 (7.71) | - | - | 39.97 (8.49) | - | - | 51.3 (10.19) | - | - |
| 10 Hrs. | 19.39 (7.57) | - | - | 39.94 (7.57) | - | - | 29.75 (8.91) | - | - |
| 20 Hrs. | 21.55 (7.33) | - | - | 18.07 (8.19) | - | - | 13.93 (9.38) | - | - |
| Control vs. 10 Hrs. | 22.04 (10.82) | 0.042 | 0.29 (0.01, 0.56) | 0.04 (11.38) | 0.997 | 0 (-0.29, 0.29) | 21.55 (13.55) | 0.113 | 0.28 (-0.07, 0.63) |
| Control vs. 20 Hrs. | 19.89 (10.67) | 0.063 | 0.26 (-0.02, 0.53) | 21.9 (11.81) | 0.065 | 0.28 (-0.02, 0.59) | 37.37 (13.86) | 0.007 | 0.48 (0.13, 0.84) |
| 10 Hrs. vs. 20 Hrs. | -2.16 (10.53) | 0.838 | -0.03 (-0.3, 0.24) | 21.86 (11.17) | 0.051 | 0.28 (0, 0.57) | 15.82 (12.97) | 0.223 | 0.21 (-0.13, 0.54) |
| UFOV2 (ms) |  |  |  |  |  |  |  |  |  |
| Control | 164.53 (14.26) | - | - | 135.73 (15.67) | - | - | 96.49 (18.66) | - | - |
| 10 Hrs. | 101.63 (14) | - | - | 103.48 (14.03) | - | - | 96.01 (16.37) | - | - |
| 20 Hrs. | 59.25 (13.63) | - | - | 111.1 (15.12) | - | - | 65.94 (17.2) | - | - |
| Control vs. 10 Hrs. | 62.9 (20) | 0.002 | 0.59 (0.21, 0.97) | 32.25 (21.02) | 0.126 | 0.3 (-0.09, 0.69) | 0.48 (24.81) | 0.985 | 0 (-0.45, 0.46) |
| Control vs. 20 Hrs. | 105.27 (19.8) | <0.001 | 0.99 (0.6, 1.37) | 24.63 (21.84) | 0.26 | 0.23 (-0.17, 0.63) | 30.55 (25.41) | 0.23 | 0.29 (-0.18, 0.76) |
| 10 Hrs. vs. 20 Hrs. | 42.37 (19.58) | 0.031 | 0.4 (0.03, 0.76) | -7.62 (20.72) | 0.713 | -0.07 (-0.45, 0.31) | 30.07 (23.81) | 0.207 | 0.28 (-0.16, 0.72) |
| UFOV3 (ms) |  |  |  |  |  |  |  |  |  |
| Control | 253.03 (14.42) | - | - | 249.29 (18.33) | - | - | 238.81 (28.58) | - | - |
| 10 Hrs. | 222.44 (13.92) | - | - | 227.36 (16.38) | - | - | 208.27 (25.08) | - | - |
| 20 Hrs. | 182.92 (13.72) | - | - | 190.06 (17.64) | - | - | 186.59 (26.44) | - | - |
| Control vs. 10 Hrs. | 30.59 (20.08) | 0.129 | 0.25 (-0.07, 0.57) | 21.93 (24.6) | 0.374 | 0.18 (-0.21, 0.57) | 30.54 (38.04) | 0.425 | 0.25 (-0.36, 0.85) |
| Control vs. 20 Hrs. | 70.11 (19.96) | 0.001 | 0.56 (0.24, 0.89) | 59.24 (25.46) | 0.021 | 0.48 (0.07, 0.88) | 52.22 (38.95) | 0.184 | 0.42 (-0.21, 1.05) |
| 10 Hrs. vs. 20 Hrs. | 39.52 (19.58) | 0.045 | 0.32 (0, 0.63) | 37.3 (24.14) | 0.124 | 0.3 (-0.08, 0.68) | 21.68 (36.47) | 0.554 | 0.17 (-0.41, 0.76) |
| UFOV4 (ms) |  |  |  |  |  |  |  |  |  |
| Control | 410.96 (12.25) | - | - | 412.05 (17.08) | - | - | 391.88 (28.92) | - | - |
| 10 Hrs. | 405.35 (11.82) | - | - | 427.12 (15.24) | - | - | 417.39 (25.42) | - | - |
| 20 Hrs. | 385.08 (11.64) | - | - | 397.33 (16.42) | - | - | 381.74 (26.85) | - | - |
| Control vs. 10 Hrs. | 5.61 (17.06) | 0.742 | 0.06 (-0.31, 0.43) | -15.07 (22.9) | 0.511 | -0.17 (-0.67, 0.33) | -25.51 (38.52) | 0.51 | -0.28 (-1.13, 0.57) |
| Control vs. 20 Hrs. | 25.88 (16.96) | 0.128 | 0.29 (-0.09, 0.66) | 14.73 (23.72) | 0.535 | 0.16 (-0.35, 0.68) | 10.14 (39.48) | 0.798 | 0.11 (-0.76, 0.98) |
| 10 Hrs. vs. 20 Hrs. | 20.27 (16.6) | 0.223 | 0.22 (-0.14, 0.59) | 29.79 (22.43) | 0.186 | 0.33 (-0.16, 0.82) | 35.65 (36.99) | 0.338 | 0.39 (-0.42, 1.21) |
| RISCAT |  |  |  |  |  |  |  |  |  |
| Control | 2.28 (0.15) | - | - | 2.08 (0.16) | - | - | 1.85 (0.19) | - | - |
| 10 Hrs. | 1.61 (0.15) | - | - | 1.83 (0.15) | - | - | 1.63 (0.17) | - | - |
| 20 Hrs. | 1.46 (0.14) | - | - | 1.64 (0.16) | - | - | 1.22 (0.18) | - | - |
| Control vs. 10 Hrs. | 0.67 (0.21) | 0.001 | 0.76 (0.28, 1.23) | 0.25 (0.22) | 0.258 | 0.28 (-0.21, 0.76) | 0.22 (0.26) | 0.395 | 0.25 (-0.32, 0.82) |
| Control vs. 20 Hrs. | 0.83 (0.21) | <0.001 | 0.93 (0.46, 1.41) | 0.44 (0.23) | 0.053 | 0.5 (-0.01, 1) | 0.63 (0.26) | 0.018 | 0.71 (0.12, 1.3) |
| 10 Hrs. vs. 20 Hrs. | 0.16 (0.2) | 0.443 | 0.18 (-0.28, 0.63) | 0.19 (0.21) | 0.372 | 0.22 (-0.26, 0.7) | 0.41 (0.25) | 0.098 | 0.46 (-0.09, 1.01) |

*Notes.* ms=milliseconds in which correctly responded; RISCAT=UFOV risk category; UFOV=Useful Field of View; Estimates from longitudinal models adjusted for baseline outcome, number of prescriptions, and Nadir CD4. Cohen's d: ~0.2 small, ~0.5 medium, ~0.8 large. P-values ≤ 0.021 are considered statistically significant at a 10% False-Discovery-Rate level.
